# Faecalibacterium langellae sp. nov. isolated from human faeces

**DOI:** 10.1099/ijsem.0.006804

**Published:** 2025-06-02

**Authors:** Adeline Ang, Véronique Robert, Atsushi Hisatomi, Masahiro Hirasaki, Shintaro Maeno, Moriya Ohkuma, Mitsuo Sakamoto, Jean-Marc Chatel, Akihito Endo

**Affiliations:** 1 Department of Nutritional Science and Food Safety, Faculty of Applied Bioscience, Tokyo University of Agriculture, Tokyo, Japan; 2 Université Paris-Saclay, INRAE, AgroParisTech, UMR1319 Micalis Institute, Jouy-en-Josas, France; 3 Microbe Division/Japan Collection of Microorganisms, RIKEN BioResource Research Center, Tsukuba, Ibaraki, Japan; 4 Research Center for Advanced Science and Innovation, Organization for Research Initiatives, Yamaguchi University, Yamaguchi, Japan; 5 NODAI Culture Collection Center, Tokyo NODAI Research Institute, Tokyo University of Agriculture, Tokyo, Japan

**Keywords:** 16S rRNA gene, butyrate-producing bacterium, *Faecalibacterium langellae*, human gut, *rpoA*, taxonomy

## Abstract

*A corrigendum of this article has been published full details can be found at 10.1099/ijsem.0.006838*

The genus *Faecalibacterium* is one of the major butyrate-producing bacteria in the human gut. Due to its production of beneficial metabolites and reduced populations in diseased patients, it is regarded as a potential biomarker of healthy gut microbiota. Strains Collection Nationale de Cultures de Microorganismes (CNCM) I-4540 and CNCM I-4541 were isolated from faeces of a healthy elderly and a healthy adult respectively. In this study, we conducted a taxonomic analysis of these strains. Phylogenetic analyses based on 16S rRNA gene and *rpoA* gene sequences revealed that the two strains belong to the genus *Faecalibacterium*. A core gene phylogenetic tree further supported their phylogenetic placement. CNCM I-4540 shared an average nucleotide identity (ANI) value of 96.7% and a digital DNA–DNA hybridization (dDDH) value of 74.3% with *Faecalibacterium taiwanense* HLW78^T^, indicating that CNCM I-4540 should be classified as *F. taiwanense*. In contrast, CNCM I-4541 did not exhibit ANI or dDDH values above the species threshold for any known species within the genus *Faecalibacterium*. These findings indicate that CNCM I-4541 represents a novel species within the genus *Faecalibacterium*, for which the name *Faecalibacterium langellae* sp. nov. is proposed. The type strain is CNCM I-4541^T^ (=CIP 112513^T^=JCM 39552^T^).

The genus *Faecalibacterium* was described in 2002 with the sole species of *Faecalibacterium prausnitzii* [1]*,* and for nearly two decades, the genus consisted exclusively of this species. This organism is recognized as one of the major butyrate producers in the gut of healthy human adults. A reduced population of the species has been reported in patients with several disorders, including Crohn’s disease, ulcerative colitis, type 2 diabetes and Covid-19 [2,4]. Consequently, the species is regarded as a potential biomarker of healthy gut microbiota and a promising candidate for the next-generation probiotics [5,6]. However, several studies over the past decade have highlighted genomic heterogeneity within the species [7,8]. Thereafter, multiple novel species within the genus *Faecalibacterium*, including *Faecalibacterium butyricigenerans, Faecalibacterium duncaniae, Faecalibacterium hattorii, Faecalibacterium longum*, *Faecalibacterium taiwanense* and *Faecalibacterium wellingii*, were described from human guts very recently, with a chicken origin of *Faecalibacterium gallinarum* [9,12].

During an ecological study of extremely oxygen-sensitive microbes in the human gut, two strains potentially belonging to the genus *Faecalibacterium* were isolated from faeces of a healthy adult and an elderly in a previous study [13]. The present study conducted a taxonomic study of the two strains and proposes a novel species classified into the genus *Faecalibacterium*.

## Bacterial strains and growth conditions

Strains CNCM I-4540 and CNCM I-4541^T^ were isolated from faeces of healthy men aged 81 and 54 years (both omnivores), respectively, in a previous study [13], and included in the taxonomic study here. Details of the isolation procedure, e.g. sample preparation, isolation medium and culture conditions, were described elsewhere [13]. *F. prausnitzii* BCRC 81047^T^ (=ATCC 27768^T^=NCIMB 13872^T^) was obtained from Bioresource Collection and Research Center (BCRC), Food Industry Research and Development Institute, Taiwan, ROC, and was used as a reference strain. Yease Casitone Fartty Acids (YCFA) broth was purged with N_2_ gas using an O_2_-removal unit (model GASCOLUMN GC-RX, Nikka Seiko) prior to autoclaving and used to culture the strains. The composition of YCFA broth was described elsewhere [14]. The strains were cultured at 37 °C for 24 h and stored at −80 °C in the presence of 20% (v/v) glycerol.

## 16S rRNA and *rpoA* gene phylogenies

16S rRNA gene sequences of CNCM I-4540, CNCM I-4541^T^ and the type strains of *Faecalibacterium* spp. were obtained from the National Center for Biotechnology Information (NCBI) database. Moreover, sequences of all copies of the 16S rRNA gene in *F. duncaniae* A2-165^T^ were obtained from its genomic data (GCA_002734145.1), since the complete genome sequence was only available for the strain among the type strains. Pairwise sequence similarities were calculated with GENETYX version 16 (GENETYX Corporation). Phylogenetic relationships among the strains tested were further studied using *rpoA* gene sequences. The complete *rpoA* gene sequences were obtained from the genome sequences of the tested strains. The genome sequences of CNCM I-4540, CNCM I-4541^T^ and the reference strains were obtained from the NCBI database, except that those of type strains of *F. butyricigenerans* and *F. longum* were obtained from the China National GeneBank Database (CNGBdb) (https://db.cngb.org/) (Table 1). Multiple alignments of the sequences with the MUltiple Sequence Comparison by Log-Expectation (muscle) and a construction of maximum likelihood (ML) phylogenetic trees were conducted using SeaView (version 5) [15]. A bootstrap analysis was performed by 100 re-samplings to estimate the confidence of tree topologies. The trees were displayed and illustrated using Interactive Tree Of Life (iTOL) [16].

**Table 1.** Genomic features of the strains used in the present study

Species	Strain	ID/assembly	Genome size (Mb)	G+C content (mol%)
*F. langellae* sp. nov.	CNCM I-4541^T^	GCF_002549775.1	2.8	58.1
*F. taiwanense*	CNCM I-4540	GCF_002549755.1	3.0	55.7
*F. taiwanense*	HLW78^T^	GCF_036632915.2	3.0	55.9
*F. prausnitzii*	ATCC 27768^T^	GCF_003324185.1	3.0	56.4
*F. duncaniae*	A2-165^T^	GCF_002734145.1	3.1	56.3
*F. hattorii*	APC922/41-1^T^	GCF_003287455.1	2.8	57.7
*F. longum*	CM04-06^T^	CNA0017731	2.9	57.8
*F. butyricigenerans*	AF52-21^T^	CNA0017730	3.0	57.5
*F. gallinarum*	JCM 17207^T^	GCF_022180365.1	2.8	59.0
*F. wellingii*	HTF-F^T^	GCF_023347535.1	2.8	56.5

**Fig. 1.**
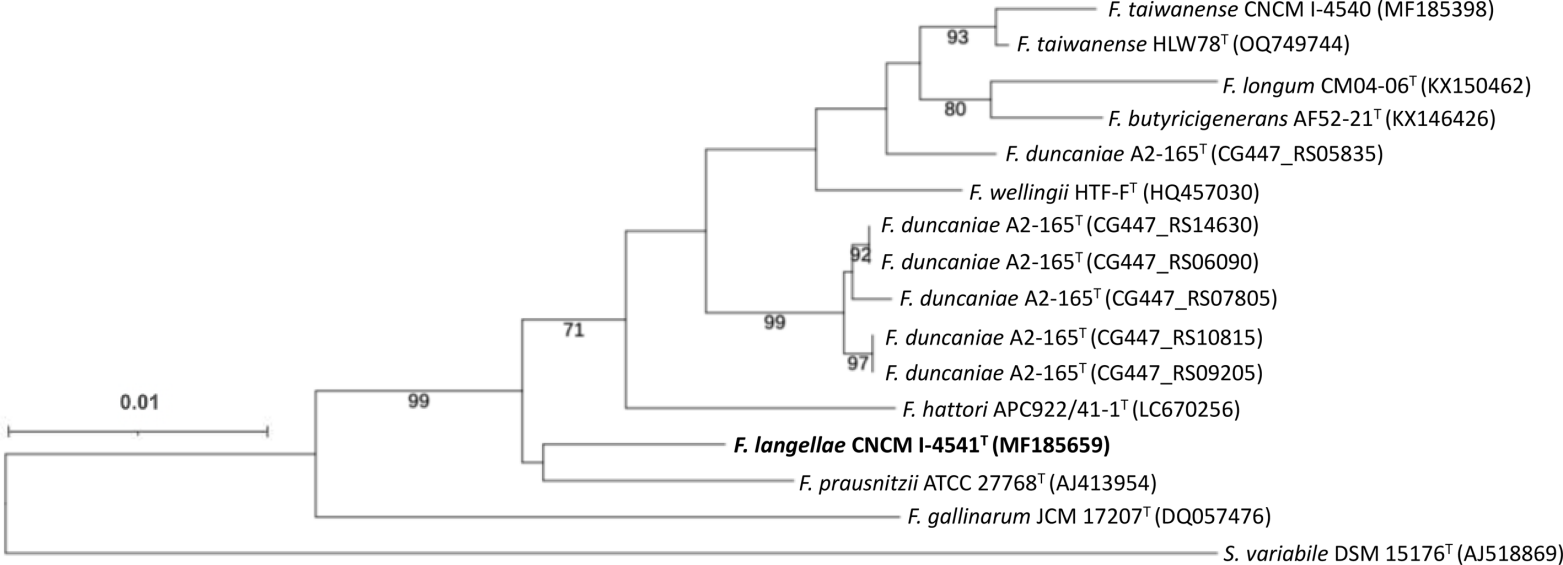
ML phylogenetic tree based on the 16S rRNA gene sequences, showing the relationship between *F. langellae* sp. nov. and related taxa. For *F. duncaniae* A2-165^T^, six copies of the 16S rRNA gene were extracted from the complete genome sequence (GCA_002734145.1), and all the copies (locus_tags) were included in this analysis. Bootstrap values (>70%) based on 100 replicates are shown at branch nodes. *Subdoligranulum variabile* DSM 15176^T^ was included as the outgroup. Bar shows 0.01 substitutions per nucleotide positions.

In 16S rRNA gene sequence analysis, CNCM I-4540 showed the highest sequence similarity of 99.1% with *F. taiwanense* HLW78^T^, followed by 98.7% with one of the six copies (CG447_RS05835) of 16S rRNA gene in *F. duncaniae* A2-165^T^ (Table 2). The highest sequence similarity of CNCM I-4541^T^ was recorded by *F. prausnitzii* ATCC 27768^T^ and CNCM I-4540 with similarity of 98.3%. The ML phylogenetic tree revealed that the two strains belonged to the *Faecalibacterium* cluster (Fig. 1). CNCM I-4540 was most related to *F. taiwanense* HLW78^T^, and CNCM I-4541^T^ was clustered with *F. prausnitzii* ATCC 27768^T^. These agreed with the result of 16S rRNA gene similarity. Of the six copies of *F. duncaniae* A2-165^T^, a copy of locus_tag CG447_RS05835 was located distantly to the remaining five copies in the tree. This was due to low sequence similarities between the sequence with locus_tag CG447_RS05835 and sequences of the remaining five copies (Table 1), and this agreed well with the previous reports [17]. Because of the low similarities among copies within a single genome, former studies suggested that the 16S rRNA gene is not a suitable gene marker for species level analysis in the genus *Faecalibacterium* [17,18].

**Table 2.** 16S rRNA gene sequence similarities among *Faecalibacterium* spp

			1	2	3	4	5	6	7	8	9	10	11	12	13	14	15
1	*F. langellae*	CNCM I-4541^T^	–	97.3	98.2	98.1	96.6	97.8	96.9	97.7	97.9	97.9	97.7	97.9	97.9	97.9	93.5
2	*F. taiwanense*	CNCM I-4540	–	–	94.7	93.9	94.1	95	96.1	93.2	95.6	92.4	92.7	92.8	92.7	92.7	92.7
3	*F. taiwanense*	HLW78^T^	–	–	–	98	97.8	97.5	97.6	97.3	98.2	97	98.6	100	99.8	99.8	99.8
4	*F. prausnitzii*	ATCC 27768^T^	–	–	–	–	97.9	97.9	97.5	97.7	97.3	98.2	97	98.4	99.8	99.7	100
5	*F. butyricigenerans*	AF52-21^T^	–	–	–	–	–	97.9	97.9	97.5	97.7	97.3	98.2	97	98.4	99.8	99.7
6	*F. hattori*	APC922/41-1^T^	–	–	–	–	–	–	97.8	97.8	97.5	97.4	97.4	98.1	97	98.7	99.8
7	*F. longum*	CM04-06^T^	–	–	–	–	–	–	–	98	97.9	97.5	97.6	97.3	98.2	97	98.6
8		A2-165^T^(CG447_RS05835)	–	–	–	–	–	–	–	–	97.9	98.7	98.7	97.4	98.8	97.1	98.1
9		A2-165^T^(CG447_RS06090)	–	–	–	–	–	–	–	–	–	97.3	98.3	97.8	96.8	98.5	97.3
10	F. duncaniae*	A2-165^T^(CG447_RS07805)	–	–	–	–	–	–	–	–	–	–	97.2	97.3	97.1	97.8	98.5
11		A2-165^T^(CG447_RS09205)	–	–	–	–	–	–	–	–	–	–	–	97.6	98.4	98.3	97
12		A2-165^T^(CG447_RS10815)	–	–	–	–	–	–	–	–	–	–	–	–	98.3	97.4	97.2
13		A2-165^T^(CG447_RS14630)	–	–	–	–	–	–	–	–	–	–	–	–	–	97.2	99.1
14	*F. gallinarum*	JCM 17207^T^	–	–	–	–	–	–	–	–	–	–	–	–	–	–	98.3
15	*F. wellingii*	HTF-F^T^	–	–	–	–	–	–	–	–	–	–	–	–	–	–	–

*All six copies of 16S rRNA gene are included for *F. duncaniae* A2-165T (GCA_002734145.1), and locus_tags of the copies are provided in parenthesis.

**Fig. 2.**
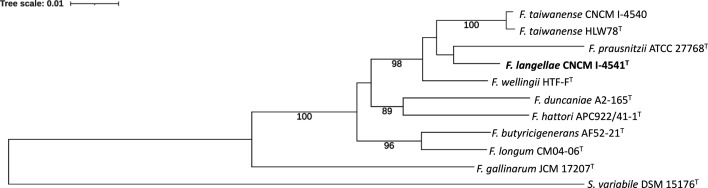
ML phylogenetic tree based on the *rpoA* gene sequences, showing the relationship between *F. langellae* sp. nov. and related taxa. Bootstrap values (>70%) based on 100 replicates are shown at branch nodes. *Subdoligranulum variabile* DSM 15176^T^ was included as the outgroup. Bar shows 0.01 substitutions per nucleotide positions.

In *rpoA* gene sequence analysis, CNCM I-4540 showed the highest sequence similarity of 99.6% with *F. taiwanense* HLW78^T^, followed by 97.1% with CNCM I-4541 (Table 3). CNCM I-4541^T^ shared the highest sequence similarity of 97.1% with CNCM I-4540 and *F. taiwanense* HLW78^T^, followed by 97.0% with *F. wellingii* HTF-F^T^ and 96.3% with *F. prausnitzii* ATCC 27768^T^. In the *rpoA* sequence-based phylogenetic tree, CNCM I-4540 clustered with *F. taiwanense* HLW78^T^, and CNCM I-4541^T^ clustered with *F. prausnitzii* ATCC 27768^T^ (Fig. 2).

**Table 3.** *rpoA* gene sequence similarities among *Faecalibacterium* spp

			1	2	3	4	5	6	7	8	9	10
1	*F. langellae*	CNCM I-4541^T^	–									
2	*F. taiwanense*	CNCM I-4540	97.1	–								
3	*F. taiwanense*	HLW78^T^	97.1	99.6	–							
4	*F. prausnitzii*	ATCC 27768^T^	96.3	95.5	95.3	–						
5	*F. butyricigenerans*	AF52-21^T^	94.2	93.7	93.7	92.7	–					
6	*F. hattori*	APC922/41-1^T^	94.5	93.3	93.4	92.4	93.9	–				
7	*F. longum*	CM04-06^T^	94.2	93.4	93.2	93.5	97.2	94.3	–			
8	*F. duncaniae*	A2-165^T^	93.8	93.9	93.8	92.9	93.7	94.8	94.5	–		
9	*F. gallinarum*	JCM 17207^T^	91.1	90.1	90.1	89.3	90.7	90.2	90.7	90.3	–	
10	*‘F. wellingii’*	HTF-F^T^	97.0	96.8	96.9	94.8	95	94.3	94.1	94.3	90.5	–

## Genome features

The genome sequences of the strains tested were obtained from databases as described above and are summarized in Table 1. Average nucleotide identity (ANI) values were determined for pairings of all strains tested based on BLAST +using PyANI (version 0.2.7) [19]. Digital DNA–DNA hybridization (dDDH) values were determined by Genome-to-Genome Distance Calculator (GGDC) version 3.0 (http://ggdc.dsmz.de/distcalc2.php) [20].

*Faecalibacterium* spp. showed a relatively narrow range of genome size (ranging from 2.8 to 3.1 Mbp) and G+C content (ranging from 55.7 to 59.0 mol%) (Table 1).

CNCM I-4540 shared the highest ANIb value of 96.7% with *F. taiwanense* HLW78^T^, which was higher than the well-accepted threshold of 94–96%, followed by 91.0% with ‘*F. wellingii*’ HTF-F^T^ and 85.2% with *F. prausnitzii* ATCC 27768^T^ (Table 4). CNCM I-4541^T^ showed the highest ANIb value of 87.5% with *F. taiwanense* HLW78*,* followed by 87.4% with ‘*F. wellingii*’ HTF-F^T^ and 85.4% with *F. prausnitzii* ATCC 27768^T^. The dDDH results agreed with the ANIb-based classification. CNCM I-4540 shared a dDDH value of 74.3% with *F. taiwanense* HLW78^T^, which was higher than the threshold of dDDH value of 70%, followed by 44.2% with ‘*F. wellingii*’ HTF-F^T^ and 29.7% with *F. prausnitzii* ATCC 27768^T^. CNCM I-4541^T^ showed the highest dDDH value of 34.1% with *F. taiwanense* HLW78^T^*,* followed by 33.8% with ‘*F. wellingii*’ HTF-F^T^ and 29.9% with *F. prausnitzii* ATCC 27768^T^, and no type strains in known *Faecalibacterium* spp. shared similarities higher than the threshold to CNCM I-4541^T^. These results indicate that CNCM I-4540 belongs to *F. taiwanense*, while CNCM I-4541^T^ does not belong to any known species of *Faecalibacterium* spp.

**Table 4.** dDDH (top, bold type) and ANIb (bottom, normal type) values (%) within the genus *Faecalibacterium*

		1	2	3	4	5	6	7	8	9	10
1	*F. langellae* CNCM I-4541^T^	–	**34.1**	**34.1**	**29.9**	**28.1**	**27.6**	**27.8**	**27.5**	**22.7**	**33.8**
2	*F. taiwanense* CNCM I-4540	87.4	–	**74.3**	**29.7**	**28.4**	**27.6**	**29.3**	**28.1**	**23.3**	**44.2**
3	*F. taiwanense* HLW78^T^	87.5	96.7	–	**30.9**	**29.3**	**28.5**	**28.3**	**29.0**	**22.8**	**45.5**
4	*F. prausnitzii* ATCC 27768^T^	85.4	85.2	85.8	–	**28.6**	**28.5**	**29.4**	**28.4**	**22.8**	**30.5**
5	*F. duncaniae* A2-165^T^	83.7	83.4	84.2	83.9	–	**29.8**	**28.3**	**28.8**	**23.1**	**28.3**
6	*F. hattorii* APC922/41-1^T^	83.8	83.2	83.9	84.1	85.1	–	**29.4**	**28.4**	**22.6**	**27.3**
7	*F. longum* CM04-06^T^	83.4	83.9	83.5	84.0	83.6	84.7	–	**41.8**	**23.3**	**27.2**
8	*F. butyricigenerans* AF52-21^T^	83.4	83.0	84.1	83.5	83.9	84.1	90.5	–	**23.9**	**28.0**
9	*F. gallinarum* JCM 17207^T^	79.0	78.7	78.0	78.8	79.0	79.0	79.4	79.4	–	**23.3**
10	*F. wellingii* HTF-F^T^	87.4	91.0	91.4	85.5	83.8	83.4	82.9	83.5	78.4	–

**Fig. 3.**
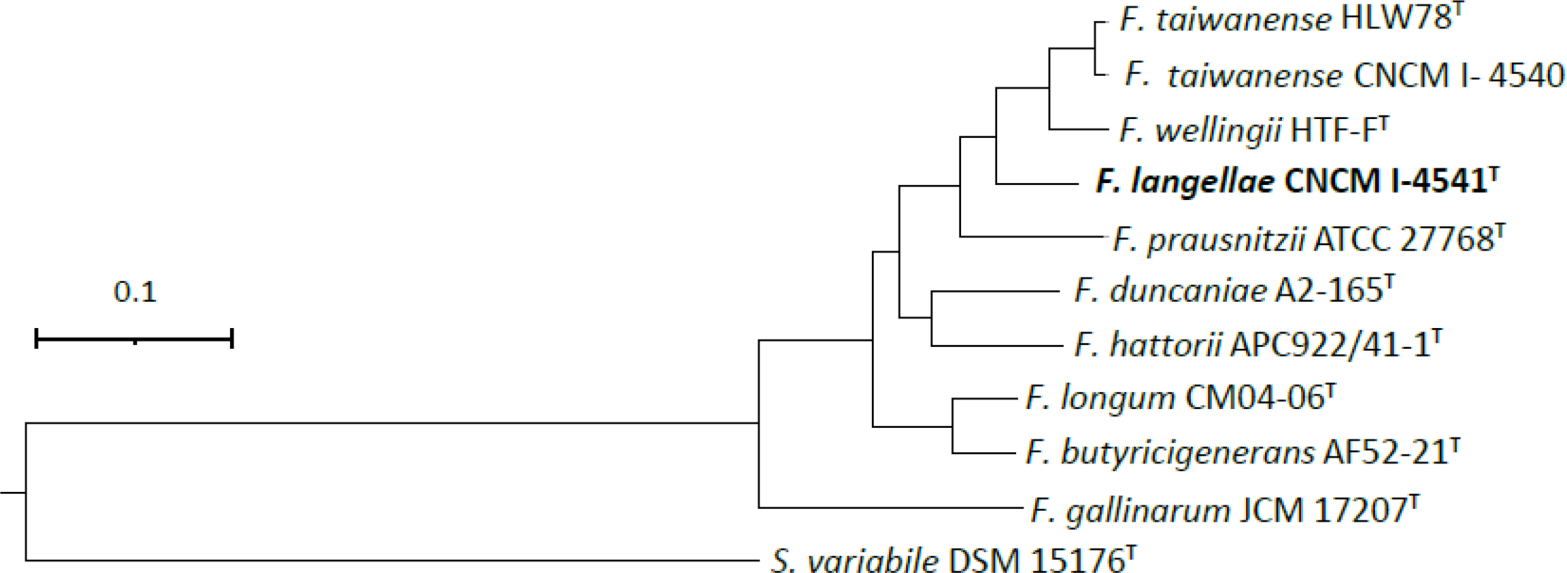
Core gene phylogenomic tree of *Faecalibacterium* spp. generated with the UBCG set by amino acid sequences. *Subdoligranulum variabile* DSM 15176^T^ was included as the outgroup. Bar shows 0.1 substitutions per positions.

Core gene phylogenetic tree was constructed with up-to-date-bacterial core gene 2 (UBCG2) set using default settings to study genome level phylogenetic relationships of strains tested [21]. CNCM I-4540 formed a cluster with *F. taiwanense* HLW78^T^ (Fig. 3). CNCM I-4541^T^ was placed in a position related to *F. prausnitzii, F. taiwanense* and *F. wellingii* with a relatively long branch. The phylogenetic relationships among *Faecalibacterium* spp. based on core genes were consistent with those based on the *rpoA* gene.

## Physiology and chemotaxonomy

Physiological and chemotaxonomic characteristics of CNCM I-4540 and CNCM I-4541^T^ were studied by the methods described by Sakamoto *et al*. [10], with an inclusion of *F. prausnitzii* BCRC 81047^T^ as a control of the phylogenetically related species. In the present study, to obtain stable growth of the strains, CNCM I-4541^T^ and *F. prausnitzii* BCRC 81047^T^ were cultured in brain heart infusion-supplemented (BHIS) broth, and CNCM I-4540 was cultured in YCFA broth. The composition of BHIS broth was previously described by Botin *et al.* [22]. The three strains grew at temperatures ranging from 30 to 42 °C, while the growth of *F. prausnitzii* BCRC 81047^T^ at 30 °C was weakly positive. They grew around pH 6.0–8.0. The strains shared slightly different carbohydrate metabolic properties (Table 5). Fucose, inositol and mannitol were weakly positive in *F. prausnitzii* BCRC 81047^T^ but negative in the remaining two strains. Gluconate was positive only in *F. prausnitzii* BCRC 81047^T^. Mannose was positive in CNCM I-4540 but showed weak reactions in the other two strains. Sucrose was positive in CNCM I-4540 and *F. prausnitzii* BCRC 81047^T^ but weak in CNCM I-4541^T^. The three strains were highly sensitive to NaCl, and the presence of 1% NaCl inhibited the growth of the strains. Enzyme activity was studied using the API ZYM system, according to the manufacturer’s instruction. The three strains were positive in acid phosphatase, naphthol-AS-BI-phosphohydrolase, β-galactosidase and β-glucosidase. Reactions in alkaline phosphatase, esterase (C 4), esterase lipase (C 8), α-galactosidase, β-glucuronidase and α-glucosidase were different among the strains (Table 5).

**Table 5.** Differential characteristics among tested strains

	1	2	3
Growth on:			
Fucose	−	−	w
Gluconate	−	−	+
Inositol	−	−	w
Mannitol	−	−	w
Mannose	w	+	w
Sucrose	w	+	+
Enzyme activity:			
Alkaline phosphatase	−	+	−
Esterase (C4)	−	+	+
Esterase lipase (C8)	−	+	−
α-Galactosidase	−	−	+
β-Glucuronidase	−	+	+
α-Glucosidase	−	−	+
Major cellular fatty acids	C16 : 0, C16 : 0 DMA, C14 : 0, C18 : 1 ω7c DMA	C15 : 0, C16 : 0, C17 : 1 ω6c	C16 : 0, C16 : 0 DMA, C18 : 1 ω7c DMA, C14 : 0
Growth at 30 °C	+	+	w

1, *F. langellae* CNCM I-4541T; 2, *F. taiwanense* CNCM I-4540; 3, *F. prausnitzii* BCRC 81047T. +, positive reaction; −, negative reaction; w, weak reaction.

Organic acids of the metabolic end products in M2G medium [1] were analysed as described elsewhere [23]. The major end products of the three strains were butyrate and formate, with slightly different ratios among strains (Table 6).

**Table 6.** End products (mM) formed after culturing of *Faecalibacterium* spp

Species	Formate	Lactate	Acetate	Butyrate
*F. langellae* CNCM I-4541^T^	8.87	−0.04	1.63	7.02
*F. taiwanense* CNCM I-4540	14.27	0.19	−0.76	13.65
*F. prausnitzii* BCRC 81047^T^	3.93	−0.23	−4.22	7.40

Values indicated with ‘−’ mean reduction of the acids after culturing of strains tested.

**Fig. 4.**
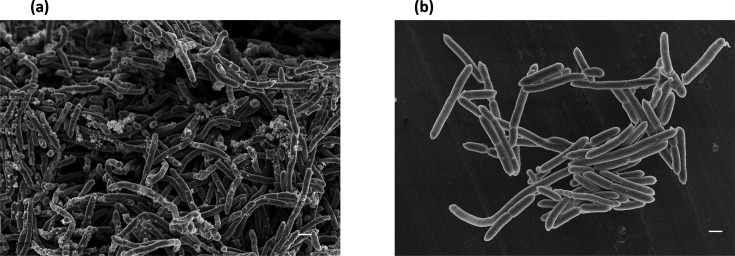
Scanning electron micrographs of cells of *F. langellae* CNCM I-4541^T^ (**a**) and *F. taiwanense* CNCM I-4540 (**b**). Bars, 1 µm.

Cells of CNCM I-4540 and CNCM I-4541^T^ were observed using scanning electron microscopy (SEM) by the method previously described by Endo *et al*. [24], with slight modifications. Samples were dried with a freeze dryer (model ES-2030, Hitachi) after fixation and dehydration and observed with a SEM (model S-4800, Hitachi). SEM images of CNCM I-4540 and CNCM I-4541^T^ are shown in Fig. 4. Cells of CNCM I-4540 and CNCM I-4541^T^ were 0.5–0.9 µm×2.0–15.0 µm and 0.5–0.9 µm×2.0–20.0 µm, respectively. Cells usually occur singly, in pairs or in short chains. These were similar to previous results of other *Faecalibacterium* spp. [10].

Based on the data provided, strain CNCM I-4541^T^ represents a novel species of the genus *Faecalibacterium*, for which the name *F. langellae* sp. nov. was proposed. Type strain is CNCM I-4541^T^=CIP 112513^T^=JCM 39552^T^.

## Description of *Faecalibacterium langellae* sp. nov.

*Faecalibacterium langellae* (lan.gel’lae. N.L. gen. masc. n. *langellae*, of Langella, named after the French microbiologist Philippe Langella, to honour his work on host-microbe interactions of beneficial bacteria, including *Faecalibacterium*)

Cells are obligately anaerobic, Gram-stain-negative and straight rods (0.5–0.9 µm×2.0–20.0 µm). Grows at 30–42 °C and at pH 6.0–8.0. Growth is inhibited in the presence of 1% (w/v) NaCl. Catalase reaction is not seen. Strain grows on cellobiose, glucose, fructose, galactose, lactose, maltose, mannose (weak) and sucrose (weak), but not on fucose, gluconate, inositol, mannitol, raffinose, ribose or xylose. Positive enzyme reactions were obtained for acid phosphatase, naphthol-AS-BI-phosphohydrolase, β-galactosidase and β-glucosidase using the API ZYM system. The major end-products in M2G medium are butyrate and formate. The major fatty acids in cells cultured in BHIS broth are C_16 : 0_, C_16 : 0_ DMA (dimethyl acetal), C_14 : 0_ and C_18 : 1_ ω7c DMA.

The type strain, CNCM I-4541^T^ (=CIP 112513^T^=JCM 39552^T^), was isolated from human faeces. The DNA G+C content of the type strain is 58.1%. The GenBank/EMBL/DDBJ accession numbers for the 16S rRNA gene sequence and the whole-genome sequence of this strain are MF185659 and GCF002549775.1, respectively.

## ABBREVIATIONS

ANI: average nucleotide identity
BCRC: Bioresource Collection and Research Center
dDDH: digital DNA–DNA hybridization
ML: maximum likelihood
SEM: scanning electron microscopy
UBGC2: up-to-date-bacterial core gene 2
