# Corrigendum: Faecalibacterium langellae sp. nov. isolated from human faeces

**DOI:** 10.1099/ijsem.0.006838

**Published:** 2025-07-04

**Authors:** Adeline Ang, Véronique Robert, Atsushi Hisatomi, Masahiro Hirasaki, Shintaro Maeno, Moriya Ohkuma, Mitsuo Sakamoto, Jean-Marc Chatel, Akihito Endo

**Affiliations:** 1 Department of Nutritional Science and Food Safety, Faculty of Applied Bioscience, Tokyo University of Agriculture, Tokyo, Japan; 2 Université Paris-Saclay, INRAE, AgroParisTech, UMR1319 Micalis Institute, Jouy-en-Josas, France; 3 Microbe Division/Japan Collection of Microorganisms, RIKEN BioResource Research Center, Tsukuba, Ibaraki, Japan; 4 Research Center for Advanced Science and Innovation, Organization for Research Initiatives, Yamaguchi University, Yamaguchi, Japan; 5 NODAI Culture Collection Center, Tokyo NODAI Research Institute, Tokyo University of Agriculture, Tokyo, Japan

In the published version of this article, there was a formatting error in the first author’s name which resulted in the surname being presented incorrectly.

The correct surname is ‘Ang’, followed by the first name of ‘Adeline’. The official arrangement of full name is Adeline Ang. The paper should therefore be cited as ‘Ang *et al*. *Int J Syst Evol Microbiol*. 2025 Jun;75 : 006804’ or “Ang A, Robert V, Hisatomi A, Hirasaki M, Maeno S, Ohkuma M, Sakamoto M, Chatel JM, Endo A. *Int J Syst Evol Microbiol*. 2025 Jun;75 : 006804’.

This has been corrected in the author list above and also in the published article.

The authors apologise for any inconvenience caused.

